# Secondhand Smoke Exposure Impairs Ion Channel Function and Contractility of Mesenteric Arteries

**DOI:** 10.1093/function/zqab041

**Published:** 2021-08-19

**Authors:** Thanhmai Le, Miguel Martín-Aragón Baudel, Arsalan Syed, Navid Singhrao, Shiyue Pan, Victor A Flores-Tamez, Abby E Burns, Kwun Nok Mimi Man, Emma Karey, Junyoung Hong, Johannes W Hell, Kent E Pinkerton, Chao-Yin Chen, Madeline Nieves-Cintrón

**Affiliations:** Department of Pharmacology, University of California Davis, Davis, CA 95616, USA; Department of Pharmacology, University of California Davis, Davis, CA 95616, USA; Department of Pharmacology, University of California Davis, Davis, CA 95616, USA; Department of Pharmacology, University of California Davis, Davis, CA 95616, USA; Department of Pharmacology, University of California Davis, Davis, CA 95616, USA; Department of Pharmacology, University of California Davis, Davis, CA 95616, USA; Department of Pharmacology, University of California Davis, Davis, CA 95616, USA; Department of Pharmacology, University of California Davis, Davis, CA 95616, USA; Department of Pharmacology, University of California Davis, Davis, CA 95616, USA; Department of Pharmacology, University of California Davis, Davis, CA 95616, USA; Department of Pharmacology, University of California Davis, Davis, CA 95616, USA; Center for Health and the Environment, University of California, Davis, CA 95616, USA; Department of Pharmacology, University of California Davis, Davis, CA 95616, USA; Department of Pharmacology, University of California Davis, Davis, CA 95616, USA

**Keywords:** Hypertension, myogenic tone, BK channel, L-type calcium channel

## Abstract

Cigarette smoke, including secondhand smoke (SHS), has significant detrimental vascular effects, but its effects on myogenic tone of small resistance arteries and the underlying mechanisms are understudied. Although it is apparent that SHS contributes to endothelial dysfunction, much less is known about how this toxicant alters arterial myocyte contraction, leading to alterations in myogenic tone. The study's goal is to determine the effects of SHS on mesenteric arterial myocyte contractility and excitability. C57BL/6J male mice were randomly assigned to either filtered air (FA) or SHS (6 h/d, 5 d/wk) exposed groups for a 4, 8, or 12-weeks period. Third and fourth-order mesenteric arteries and arterial myocytes were acutely isolated and evaluated with pressure myography and patch clamp electrophysiology, respectively. Myogenic tone was found to be elevated in mesenteric arteries from mice exposed to SHS for 12 wk but not for 4 or 8 wk. These results were correlated with an increase in L-type Ca^2+^ channel activity in mesenteric arterial myocytes after 12 wk of SHS exposure. Moreover, 12 wk SHS exposed arterial myocytes have reduced total potassium channel current density, which correlates with a depolarized membrane potential (*Vm*). These results suggest that SHS exposure induces alterations in key ionic conductances that modulate arterial myocyte contractility and myogenic tone. Thus, chronic exposure to an environmentally relevant concentration of SHS impairs mesenteric arterial myocyte electrophysiology and myogenic tone, which may contribute to increased blood pressure and risks of developing vascular complications due to passive exposure to cigarette smoke.

## Teaser

Secondhand smoke alters vascular smooth muslce ion channel function and vascular tone. Figure created with BioRender.com

## Introduction

Exposure to cigarette smoke (CS) is a major cause of cardiovascular complications, including stroke and coronary and peripheral artery diseases.^1^^,^^2^ Importantly, even passive exposure to CS aerosols (ie secondhand smoke, SHS) significantly elevates lifelong cardiovascular risk.^3^ Despite widespread national and local media campaigns, SHS continues to be a prevalent indoor pollutant,^4–7^ with deleterious vascular effect.^2^^,^^8^ Whereas the multifactorial nature of changes in the vasculature is well-established (eg endothelial dysfunction and aberrant central control regulation),^2^^,^^9^^,^^10^ the mechanisms contributing to this, including impairment of arterial myocytes function, remains poorly studied.

The contractile state of arterial myocytes (myogenic tone) in small resistance arteries and arterioles is a key determinant of arterial diameter, and thus exert a major influence on organ perfusion, total peripheral resistance, and blood pressure.^11^^,^^12^ Myogenic tone regulation relies on the dynamic interplay between different ionic conductance that helps controlling arterial myocyte membrane potential and intracellular calcium concentration ([Ca^2+^]_i_).^13^ Ca^2+^ influx via L-type Ca^2+^ channels (LTCC) in the plasma membrane of arterial myocytes leads to contraction.^13^ In contrast, the activity of K^+^ channels provides negative feedback regulation of LTCC activity, thereby regulating [Ca^2+^]_i_, myocyte contractility, and the level of myogenic tone.^14^^,^^15^ Intriguingly, exposure to SHS has been suggested to alter arterial constriction.^16^ In addition to endothelial dysfunction,^8^^,^^17^ reduced brachial artery relaxation in response to nitroglycerin has been observed in people exposed to CS.^18^ These data suggest cigarette aerosols can impair arterial myocyte function, which may contribute to tobacco-related vascular complications. Yet how SHS alters arterial myocyte function and whether this contributes to altered myogenic tone is unclear.

In this study, we test the hypothesis that arterial myocyte contractility of mesenteric small resistance arteries is altered in mice exposed to SHS. The effects of SHS on vascular contractility and myocyte function were systematically evaluated in freshly isolated small resistance arteries and arterial myocytes from mice exposed to environmentally relevant concentrations of SHS (3 mg/m^3^ total suspended particles, TSP). This concentration was selected to reflect real-life fine particulate matter concentration measured in bars that permit smoking.^19–21^ Our data reveal a time-dependent effect of SHS exposure on basal arterial tone, with higher myogenic tone in mesenteric arteries of mice exposed to SHS for 12 wk, but not at earlier time points of 4 and 8 wk. This change in tone was correlated with enhanced LTCC function, reduced K^+^ channel activity and depolarized membrane potential in 12-week SHS-exposed mesenteric arterial myocytes. Moreover, arterial myocytes from SHS exposed mice showed increase activity of the transcription factor of activated T cells (NFAT). These results highlight previously unappreciated changes in key ionic conductances that alter arterial myocyte excitability and myogenic tone in response to environmentally relevant concentration of SHS. These SHS-induced changes in arterial myocyte excitability and contractility may contribute to changes in blood pressure and cardiovascular risks observed in nonsmokers exposed to SHS.

## Methods

### Animals

All animal-related procedures were performed in strict compliance with protocols approved by the Institutional Animal Care and Use Committee at the University of California, Davis. C57BL/6J male mice, 8-week-old, were obtained from The Jackson Laboratory and housed individually in polycarbonate cages with temperature control (21 ± 2°C) and on a 12-hour light/dark cycle. Mice were allowed to acclimatize to the facility for 2 wk before beginning the exposures. At 10 wk of age whole body exposure to SHS was carried out in exposure chambers at the Center for Health and the Environment, University of California, Davis. Mice had *ad libitum* access to water and food.

### Secondhand Smoke Exposure

Adult male C57BL/6J mice were randomly assigned to filter air (FA; control group) or SHS exposed groups. Side stream CS from humidified 3R4F research cigarettes (Tobacco Health Research Institute; Lexington, KY) produced by a smoking apparatus built at the Inhalation Exposure Core facility was used as the surrogate to SHS.^22^ An automatically metered puffer smoked the cigarettes under Federal Trade Commission conditions (35 mL puff, 2 s duration, and 1 puff per min). The exposures were characterized for three major constituents of SHS: Nicotine, carbon monoxide (CO), and total suspended particulates (TSP; Table 1). The target concentration of smoke was set at 3 mg/m^3^ TSP. This concentration was selected based on the average real-life fine particulate matter concentration in smoking restaurants and bars, which has been reported to range from 0.2–0.6 mg/m^3^ with the maximal level reaching 3 mg/m^3^.^19–21^

**Figure 1.**
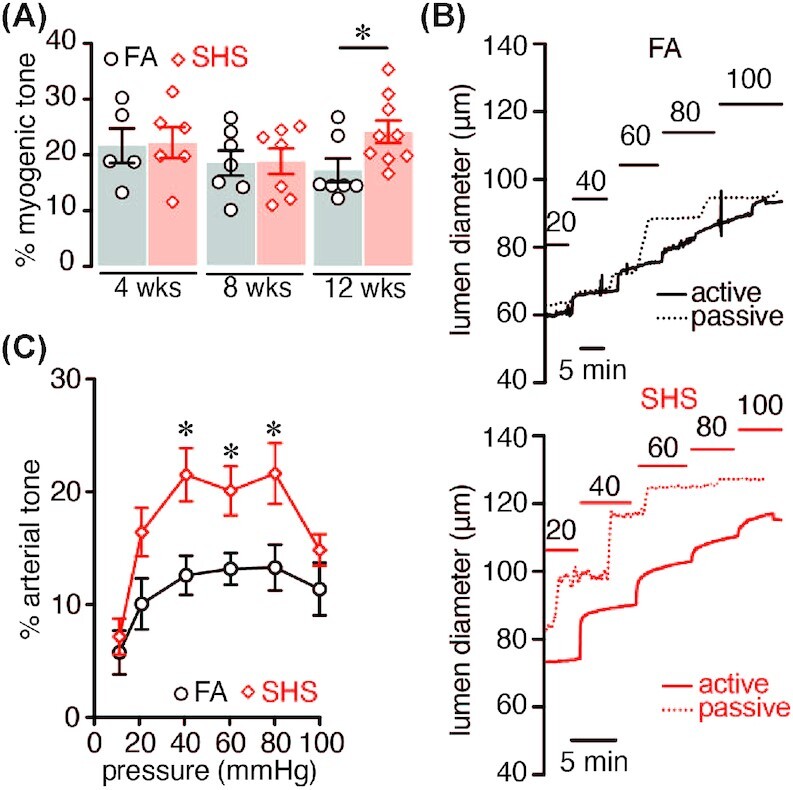
Myogenic tone in mice exposed to FA or SHS. (A) Percentage myogenic tone at 60 mmHg after 4, 8, and 12 wk in FA (*n* = 5/5, 7/5, 7/4 arteries/mice for each respective group) and SHS (*n* = 6/5, 7/5, 9/5 arteries/mice for each respective group) exposed mice (*P < .05 two-way ANOVA). (B) Representative pressure myography traces obtained at specified intravascular pressures from FA and 12 wk SHS arteries. (C) Myogenic tone–pressure curves of FA (*n* = 7 arteries; 6 mice) and 12 wk SHS-exposed arteries (*n* = 9 arteries; 7 mice). *P < .05, one-way ANOVA, Bonferroni post-test.

**Table 1.** Exposure Conditions

Component	Average	SD
Nicotine (mg/m^3^)	0.82	0.32
TSP (mg/m^3^)	3.02	0.06
Carbon Monoxide (ppm)	15.16	1.12

### Myograph Experiments

Freshly dissected third and fourth order mesenteric arteries (average diameter 90–120 μm) were cannulated on glass micropipettes and mounted on a 5 mL myograph chamber (Living Systems Instrumentations). A PS-200 pressure Servo controller with peristaltic pump (Living Systems Instrumentations) was used in the pressure mode to control and monitor the intravascular pressure in the cannulated vessel. One cannula is connected to a servo-pump while the other is closed to flow with a valve. The pressure is sensed by a pressure transducer positioned in line with the cannulated vessel. Prior to experiments, arteries were pressurized to 20 mmHg and allowed to equilibrate while perfused continuously with physiological buffer (37°C, 30 min, and 3–5 mL/min) of the following composition (in mM): 119 NaCl, 4.7 KCl, 2 CaCl_2_, 24 NaHCO_3_, 1.2 KH_2_PO_4_, 1.2 MgSO_4_, 0.023 EDTA, and 10 D-glucose. The pH of the solution was maintained at 7.4 by aerating with a 95% O_2_/5% CO_2_ balanced gas mix. An IonOptix system was used for data acquisition, followed by analysis with IonOptix software v6.6. Following equilibration, arterial viability was tested by examining the contraction elicited by 60 mM KCl. Only arteries that constricted robustly to the high-K^+^ solution were used for experiments (Table 1). Viable arteries were pressurized to 60 mmHg and allowed to develop stable myogenic tone. Arteries that didn't maintain tone after 1 h were discarded. After stable tone was established, arteries were experimented upon as described throughout the manuscript. Myogenic tone data are presented as the active diameter at a given intraluminal pressure relative to the maximally dilated diameter obtained at that same pressure in a Ca^2+^ free solution that also contained the L-type Ca^2+^ channel blocker nifedipine (1 µM).

### Arterial Myocyte Isolation

Mesenteric arteries were dissected in ice-cold dissection buffer comprised of (in mM): 140 NaCl, 5 KCl, 2 MgCl_2_, 10 D-glucose, and 10 HEPES, pH 7.4, with NaOH. Arteries were incubated (7 min, 37°C) in dissection buffer supplemented with papain (1 mg/mL) and dithiothreitol (1 mg/mL), followed by incubation in dissection buffer containing collagenase type H (1.7 mg/mL), elastase (0.5 mg/mL), and trypsin inhibitor (1 mg/mL; 37°C and 10 min). After digestion, arteries were washed with ice-cold dissection buffer. Single smooth muscle cells were obtained by gently pipetting the arteries up and down with fire-polished glass pipettes of decreasing size. Cells were kept in ice-cold dissection buffer until used.

### Electrophysiology

All experiments were carried-out at room temperature (23–25°C). An Axopatch 200B amplifier and Digidata 1440 digitizer (Molecular Devices) was used for data acquisition. Single L-type Ca^2+^ currents were recorded in freshly isolated arterial myocytes in the cell-attached mode. Signals were recorded at 10 kHz and low pass filtered at 2 kHz. Patch pipettes were made from borosilicate capillary glass (1.17 OD) using a P97 flaming micropipette puller (Sutter Instruments) and fire polished with a World Precision instrument polisher. Pipette resistance was maintained at 6–7 MΩ. Cells were perfused with a high K^+^ solution composed of (mM): 145 KCl, 10 NaCl, and 10 HEPES, pH 7.4 (KOH). This high K^+^ extracellular solution zeroed the patch transmembrane potential. The pipette solution consisted of (mM) 20 tetraethylammonium chloride (TEA-Cl), 110 CaCl_2_, and 10 HEPES, pH 7.3 (TEA-OH). The pipette solution was supplemented with (S)-(-)-BayK-8644 (500 nM) to promote longer open times and resolve channel openings as previously performed by our group and others.^23–25^ Single-channel events were recorded during a 2 second single pulse protocol to 0 mV from a holding potential of −80 mV every 5 s. An average of 50 sweeps were collected with each recording file. Single L-type channel activity was measured as n*Po*, where n is the number of channels and *Po* is the open probability, using the half-amplitude event detection algorithm of pClamp 10. We corrected the n*Po* by the number of channels (*n*) providing open probability and calculating the mean ensemble average current. We used a binary coupled Markov chain model to simulate and fit independent membrane current records of partially coupled channels. The program was written in MATLAB^®^ language. A half-amplitude protocol, with the unitary event level set at 0.5 pA was used to identify channel openings. Ca_V_1.2 activity was modeled as a first order, discrete Markov chain, the specific Markovian transition matrix was estimated from the current records and the corresponding channel open time courses using the built-in Hidden Markov parameter estimation function on MATLAB^®^. The transition matrix was modeled as a partially coupled Markov chain with three parameters. A coupling coefficient parameter (κ) is the coupling coefficient between fully uncoupled and fully coupled events. A channel open-to-open probability parameter (ρ) and channel closed-to-closed probability (ς). Together, these parameters describe the contribution of the fully uncoupled case to the transition matrix. For each record, the optimum set of parameters (κ, ρ, and ς) for the partially coupled Markov chain model was fitted using a gradient descent algorithm.

Whole-cell potassium currents (I_K_) from freshly dissociated arterial myocytes were evoked by 0.6s depolarizing pulses from a holding potential of −70 mV to +80 mV in increments of 10 mV. Extracellular solution consisted of (mM): 134 NaCl, 6 KCl, 1 MgCl_2_, 2 CaCl_2_, 7 glucose, 10 HEPES, and pH 7.4 (NaOH). The intracellular solution contained (mM): 140 KCl, 8 NaCl, 10 EGTA, 10 HEPES, 1 NaGTP, 0.02 CaCl_2_, 3 MgATP, and pH 7.2 (KOH). Currents were recorded under control condition and after application of 100 nM IBTx (mixed in the bath solution) to obtain the IBTx-sensitive component (I_BK_) of the I_K_. Currents were sampled at 20 kHz and low pass filtered at 5 kHz. Recordings were analyzed using pCLAMP10 analysis software. Membrane voltage (*V_M_*) was recorded under the current-clamp configuration using the perforated patch modality and a gap-free protocol. The internal solution for these experiments consisted of (in mM): 40 KCl, 95 K glutamate, 8 CaCl_2_, and 10 HEPES (pH 7.2). The pipette solution was supplemented with Amphotericin B (0.24 mg/mL). The bath solution was the same as that used for whole-cell recordings.

### Immunoblotting

Mouse mesenteric arteries were homogenized in a RIPA lysis buffer solution (mM): 50 Tris base, 150 NaCl, 1 EDTA, 1% Triton X-100, 0.04% sodium dodecyl sulfate (SDS) with protease inhibitors (1 µg/mL pepstatin A, 10 µg/mL leupeptin, 20 µg/mL aprotinin, and 200 nM phenylmethylsulphonyl fluoride). The lysate was cleared by centrifugation (250 000 × *g*, 30 min, 4°C), the supernatant was used as the arterial lysate. The samples were heated on Laemmli sample buffer (Bio-Rad) for 20 min at 65°C. SDS-polyacrylamide gel electrophoresis (150–200 V: 1h) using a precast 4%–20% gradient gel (Bio-Rad) was used to resolve the proteins. After SDS separation, proteins were transferred to a polyvinylidene difluoride membrane (50 V, 1200 min, and 4°C). A 5% non-fat dry milk solution in tris-buffered saline with 0.05% Tween 20 (TBS-T) was used as a blocking solution. Membranes were blocked in this solution for 1 h at room temperature before incubation with the primary antibody. The Ca_V_1.2 antibody (9.6 µg/mL of FP1 rabbit antiCa_V_1.2^26^ was diluted in 5% nonfat dried milk TBS-T. This antibody has been extensively validated.^27^ Membranes were incubated with primary antibody overnight at 4°C, followed by exposure to horseradish peroxidase conjugated goat antirabbit secondary antibody (HRP; Invitrogen, catalog number 31460) at 1:5000 dilution in 5% nonfat dry milk TBS-T). Chemiluminescence reagents Femto (Thermo Fisher Scientific) was used to visualize the bands. Quantification of band intensity was done with the Image J software (NIH) or Image lab version 6.0.1 from Bio-Rad.

### Proximity Ligation Assay (PLA)

The Duolink probe maker kit was used to tag a single monoclonal Ca_V_1.2 antibody (Antibodies Inc; catalog number 75–257; clone N263/31) with either PLUS or MINUS probes. Briefly, two aliquots of 20 µL (1 mg/mL concentration) of monoclonal antiCa_V_1.2 antibody were tagged with either plus (DUO92009-1KT) or minus (DUO92010-1KT) probe per manufacturer instructions. The PLUS or MINUS tagged antiCa_V_1.2 antibody was used for PLA assay. Acutely dissociated myocytes were plated in glass coverslips and allowed to sit for 30 min at room temperature. Cells were fixed in glyoxal (Sigma-Aldrich catalog number 128465) solution (20 min, RT),^28^ and quenched in 100 mM glycine in PBS (15 min) followed by 2×3 min washes with phosphate-buffered saline (PBS) solution containing (in mM) 138 NaCl, 3 KCl, 10 Na_2_HPO_4_, 2 NaH_2_PO_4_, 5 D-glucose, 0.1 CaCl_2_, 0.1 MgSO_4_, and pH 7.4 (NaOH). For permeabilization, cells were incubated in 0.1% Triton-100 solution in PBS (30 min, RT). After permeabilization, cells were blocked with Duolink Blocking Solution (1 h, RT). For Ca_V_1.2 labeling, cells were incubated overnight with the following antibody combination: Mouse antiCa_V_1.2-Plus and antiCa_V_1.2-Minus (1:200). As a negative control, cells were incubated with either mouse antiCa_V_1.2-Plus, antiCa_V_1.2-Minus or untagged antiCa_V_1.2. The antibodies were diluted in Duolink Antibody Diluent Solution as per manufacturer instructions. After overnight incubation with probe-tagged primary antibody, cells were washed with Duolink Buffer A (3 × 5 min) followed by incubation (30 min 37°C) with the ligation solution. The ligation solution was removed, and cells were washed 3x for 3 min. After the final wash, the cells were incubated in amplification solution (120 min 37°C) followed by 2×10 min washes with buffer B and 1×1min wash in 0.01% buffer B in dH_2_O. For detection of PLA puncta between phosphorylated NFAT and total NFAT, cells were incubated overnight with antiNFAT4 (Thermo Fisher: catalog number AF5834SP) and antiphospho-NFAT (Sigma-Aldrich; catalog number SAB4503947). Overnight incubation with primary antibodies was followed by 1 h incubation (37°C) with antigoat-plus and antirabbit-minus probes. The ligation and amplification steps proceeded as explained above.

### Quantitative PCR

Mesenteric arteries were collected and kept in RNA*later* solution (Invitrogen). Total RNA was isolated with the SPLIT RNA Extraction kit (Lexogen) following manufacturer instructions and reverse transcribed with Superscript (Invitrogen). The reverse transcription products were used for quantitative PCR using the TaqMan reagents (Applied Biosystems). Specific primers to detect BKα1 (assay ID Mm01268569_m1) and BKβ1 (assay ID: Mm00466621_m1) subunits and GAPDH (Mn99999915_g1) were acquired from ThermoFisher. An Applied Biosystems real-time PCR instrument was used for amplification. GAPDH was used as an internal control.

### Statistics

GraphPad Prism software was used to analyze the data. The data are reported as mean ± SEM. A normality test was run prior to analysis, and statistical significance was determined using Student's *t*-test, nonparametric test, or one or two-way ANOVA for multiple comparisons followed by the appropriate posthoc test. *P*-values of less than .05 was considered statistically significant (indicated by an asterisk in the figures).

## Results

C57BL/6J mice were randomized into FA and SHS exposed groups for 4-, 8-, and 12-weeks period. Table 1 summarizes the exposure conditions. Mice randomization was performed by a third party at the exposure facility; experimenters were blinded to the experimental conditions during data collection and analysis. Freshly isolated mesenteric, small resistance arteries and arterial myocytes from mice exposed to FA and SHS were used to assess the effects of SHS on arterial contractility and smooth muscle electrophysiology.

### SHS Increases Myogenic Tone in Mesenteric Arteries

Following 4, 8, and 12 wk of FA or SHS exposure, pressure-induced constriction was assessed in freshly isolated third and fourth order mesenteric murine arteries. Arteries were pressurized at 60 mmHg and allowed to develop tone. Peak 60 mM K^+^-induced constriction was similar between all groups tested (Table 2). No significant difference in tone was observed in arteries from mice exposed to SHS after 4 and 8-weeks, compared to arteries from FA mice (Figure 1A and Table 2). However, a significant increase in tone was detected after 12 wk of SHS exposure compared to FA arteries (Figure 1A and Table 2). These results suggest a time-dependent effect of SHS exposure in altering mouse mesenteric myogenic tone.

**Table 2.** Myogenic Tone in Mesenteric Arteries from Mice Exposed to FA or SHS for 4, 8, and 12 wk

% Myogenic Tone at 60 mmHg		
Exposure time	FA	SHS
4 wk	21.6 ± 3.1 (*n* = 5)	22.15 ± 2.8 (*n* = 6)
8 wk	18.42 ± 2.2 (*n* = 7)	18.69 ± 2.3 (*n* = 7)
12 wk	17.30 ± 2.1 (*n* = 7)	24.20 ± 2.1* (*n* = 9)
**Peak 60 mM K^+^ constriction (%)**		
4 wk	79.3 ± 4.2	73.5 ± 4.9
8 wk	67.32 ± 3.0	70.1 ± 4.6
12 wk	63.37 ± 5.2	69.8 ± 4.0

Values are mean ± SEM. * *P* < .05 with two-way ANOVA test for comparison between FA and SHS datasets.

To further probe SHS effects on myocyte contractility, we evaluated the myogenic response at a range of pressures (10–100 mmHg) in a cohort of mice exposed to FA or SHS for 12 wk. The peak 60 mM K^+^ constriction was similar between FA and SHS arteries (Table 3). However, mesenteric arteries from mice exposed to SHS for 12 wk showed significantly elevated myogenic tone at several intravascular pressures (20–80 mmHg) compared to FA arteries (Figure 1B and C,  Table 3). These results indicate that chronic SHS exposure alters myogenic tone in a physiologically relevant pressure range, and this may contribute to vascular complications in individuals exposed to these aerosols. Given that the most dramatic change in myogenic tone was observed at 12 wk of SHS exposure, all subsequent experiments were performed on tissues collected from mice at this exposure timepoint.

**Table 3.** Pressure–Tone Values for Mesenteric Arteries from Mice Exposed to FA and SHS for 12 wk

	FA	SHS
**Peak 60 mM K^+^ constriction (%)**	64.4 ± 3.0	67.9 ± 4.0
**% myogenic tone at the specified intravascular pressure**		
10 mmHg	7.0 ± 1.9	8.3 ± 1.6
20 mmHg	10.6 ± 2.5	17.6 ± 2.1*
40 mmHg	13.0 ± 2.1	24.0 ± 2.1*
60 mmHg	13.9 ± 1.8	22.3 ± 2.0*
80 mmHg	14.4 ± 2.5	23.8 ± 2.7*
100 mmHg	13.1 ± 2.9	16.0 ± 1.6

Values are mean ± SEM. FA (*n* = 7 arteries); (SHS *n* = 9). * *P* < .05 with one-way ANOVA, Bonferroni post-test.

### Elevated LTCC Activity in Chronic SHS-Exposed Arterial Myocytes

**Figure 2.**
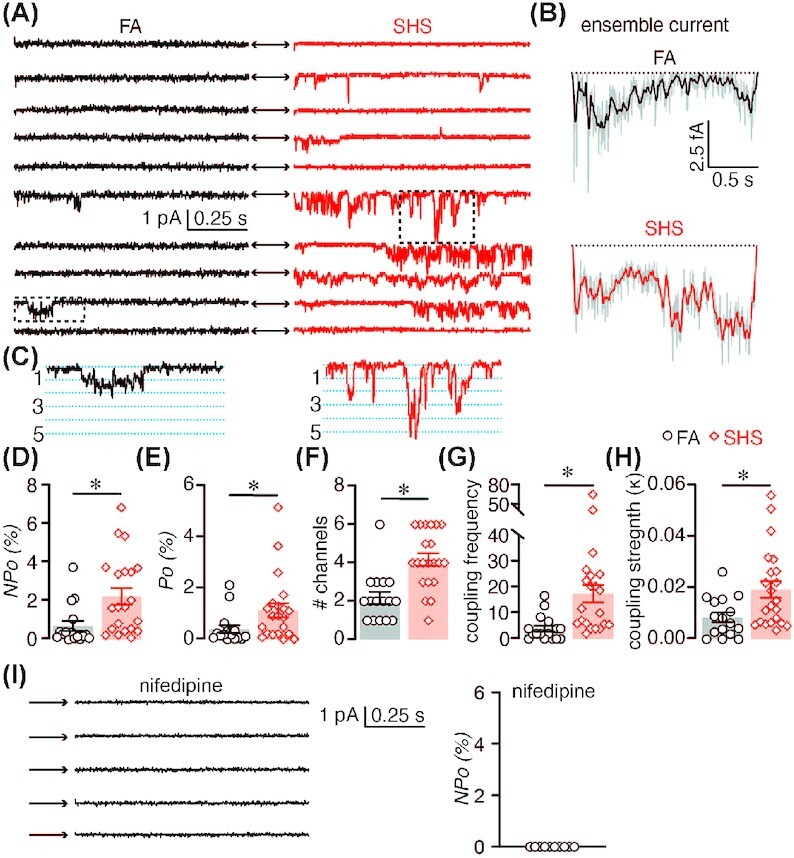
LTCC activity in FA and chronic SHS-exposed arterial myocytes. (A) Representative consecutive LTCC single-channel traces recorded from FA and 12 wk SHS-exposed arterial myocytes and (B) corresponding ensemble average currents. (C) Magnified region from the dotted squares in panel A showing coupled channel events. (D–H) Scatter plot of the mean ± SEM for *nP_o_* (D), *P_o_* (E), # channels (F), coupled frequency (G), and coupled strength (H) of LTCC single-channel data from FA (*n* = 16 cells) and 12 wk SHS-exposed (*n* = 21 cells) arterial myocytes. *P < .05 compared to FA, Mann–Whitney. (I) Representative consecutive LTCC single-channel traces in presence of the LTCC channel blocker nifedipine (1 μM).

Next, we proceeded to examine mechanisms underlying elevated mesenteric myogenic tone in chronic SHS-exposed mice. As the primary source of Ca^2+^ entry in arterial myocytes, LTCCs activity have profound effects on arterial myocyte contractility and myogenic tone.^13^ Indeed, several studies show that increased LTCC activity and expression leads to altered myogenic tone in animal models of hypertension and diabetes.^29^ Thus, we aimed to determine the effects of chronic SHS on LTCC function in arterial myocytes from mice exposed to FA and chronic SHS. For this, we used the cell-attached configuration of the patch-clamp technique to measure single-channel LTCC activity. Qualitative analysis of sequential single-channel traces and ensemble currents suggest that LTCC activity is augmented in chronic SHS-exposed arterial myocytes relative to age-matched FA smooth muscle cells (Figure 2A–C).

Comprehensive analysis of the single-channel data shows a three-fold increase in the n*P_o_* (*n* = number of channels in the patch and *P_o_ = *open probability of the channels) of LTCC in arterial myocytes from chronic SHS-exposed mice (n*P_o_ *= 2.3 ± 0.4) compared to cells from FA mice (n*P_o_* = 0.72 ± 0.3; Figure 2D). By estimating the number of LTCCs in the patch, (based on simultaneous channel openings over the depolarizing step) to calculate the LTCC open probability, we found a similar increase in channel *P_o_* in cells from chronic SHS-exposed mice compared to myocytes from FA mice (0.4 ± 0.2 for FA and 1.2 ± 0.3 for SHS; *P* < .05). We also observed an increase in the number of LTCCs in the patch of chronic SHS-exposed cells (Figure 2F). Closer inspection of the single-channel traces suggest that several events can be produced by the simultaneous opening and closing of two or more LTCCs (Figure 2C) in a gating mode known as cooperative gating.^24^^,^^30^^,^^31^ Because of the oligomerization of two or more channels working in unison at the plasma membrane, this gating modality amplifies Ca^2+^ influx, and thus may influence arterial myocyte contractility.^24^^,^^31^ Quantitative examination of this gating modality revealed increased frequency (Figure 2G) and strength (Figure 2H) of coupled events in chronic SHS-exposed arterial myocytes compared to FA cells. These results indicate an increase in LTCC activity and cooperative gating in arterial myocytes from mice chronically exposed to SHS.

### Increased Ca_V_1.2 Expression and Clustering in Chronic SHS-Exposed Arterial Myocytes

**Figure 3.**
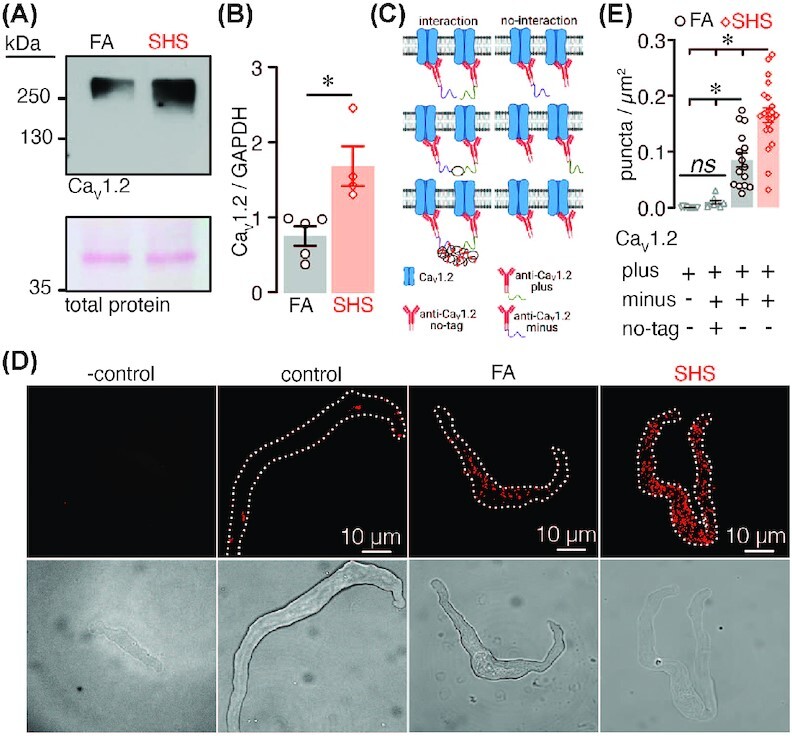
LTCC expression and oligomerization in FA and chronic SHS-exposed samples. (A) Representative WB showing immunoreactive bands for the LTCC pore forming subunit Ca_V_1.2 and total protein in mesenteric artery lysates from FA and SHS-exposed mice. (B) Scatter plot of densitometry for Ca_V_1.2 protein abundance normalized to GAPDH (*n* = 4–5 lysates per condition; *P* < .05, Mann–Whitney). (C) Cartoon of PLA approach. (D) Representative fluorescence PLA (top) and differential interference contrast (bottom) images of FA and 12 wk SHS-exposed arterial myocytes labeled with the Ca_V_1.2 plus and Ca_V_1.2 minus probes. In some experiments one of the probes was left out as negative control. We also used an excess of un-tagged Ca_V_1.2 antibody as an additional control. (E) Quantification of PLA puncta per cell area (puncta/µm^2^) in FA (*n* = 15 cells) and 12 wk SHS-exposed (*n* = 21 cells) arterial myocytes, as well as control cells (*n* = 8 cells) and negative controls (*n* = 12 cells). *P* < .05, two-way ANOVA, followed by Tukey's multiple comparison post-test.

To examine mechanisms underlying the increase in LTCC activity and cooperative gating in chronic SHS-exposed myocytes, we first examined total expression of the LTCC pore-forming alpha subunit Ca_V_1.2 in FA and chronic SHS-exposed arterial lysates using western blot (WB) analysis. This analysis revealed a significant increase in total Ca_V_1.2 expression in chronic SHS-exposed arterial lysates compared to FA lysates (Figure 3A). These results correlate with the significant elevation in the number of LTCCs in the patch described above (see Figure 2F).

Cooperative gating of LTCCs is thought to be dependent on physical proximity between channels.^24^^,^^30–32^ To define whether an increased association of Ca_V_1.2 subunits at the plasma membrane of arterial myocytes underlies the elevation in cooperative gating of LTCCs in chronic SHS-exposed myocytes, we used a modified proximity ligation assay (PLA) approach. For this, a mouse monoclonal antiCa_V_1.2 primary antibody was tagged in-house with the PLA plus or minus probes (Figure 3C). The monoclonal antiCa_V_1.2-plus and antiCa_V_1.2-minus antibodies should bind to different Ca_V_1.2 subunits. If these individual Ca_V_1.2 subunits are within 40 nm of each other, a PLA punctum should be produced.^33^^,^^34^ This outcome will suggest the close association between at least two Ca_V_1.2 subunits. In freshly dissociated arterial myocytes labeled with antiCa_V_1.2-plus and antiCa_V_1.2-minus primary antibodies, data showed a significant increase in PLA puncta in chronic SHS-exposed cells compared to FA cells (Figure 3C and D). The Ca_V_1.2-plus and Ca_V_1.2-minus antibodies bind randomly to the LTCC Ca_V_1.2 subunits, thus as an additional control we included an excess of untagged antiCa_V_1.2 primary antibody together with antiCa_V_1.2-plus and antiCa_V_1.2-minus antibodies. Note that inclusion of an excess of untagged antiCa_V_1.2 primary antibody significantly decreased the number of puncta as the untagged antibody is competing for the same binding site as the plus/minus Ca_V_1.2 antibodies. Moreover, no PLA signal was observed when FA and chronic SHS cells were treated with only one primary antibody (Figure 3C and D). These results suggest a close association between Ca_V_1.2 subunits as a consequence of chronic SHS in arterial myocytes. Moreover, data indicate an upregulation in protein abundance and increased proximity of Ca_V_1.2 subunits, which may contribute to elevated LTCC activity and frequency of cooperative gating events in chronic SHS-exposed arteries/arterial myocytes.

### Chronic SHS Alters Arterial Myocyte V_M_ and I_K_

**Figure 4.**
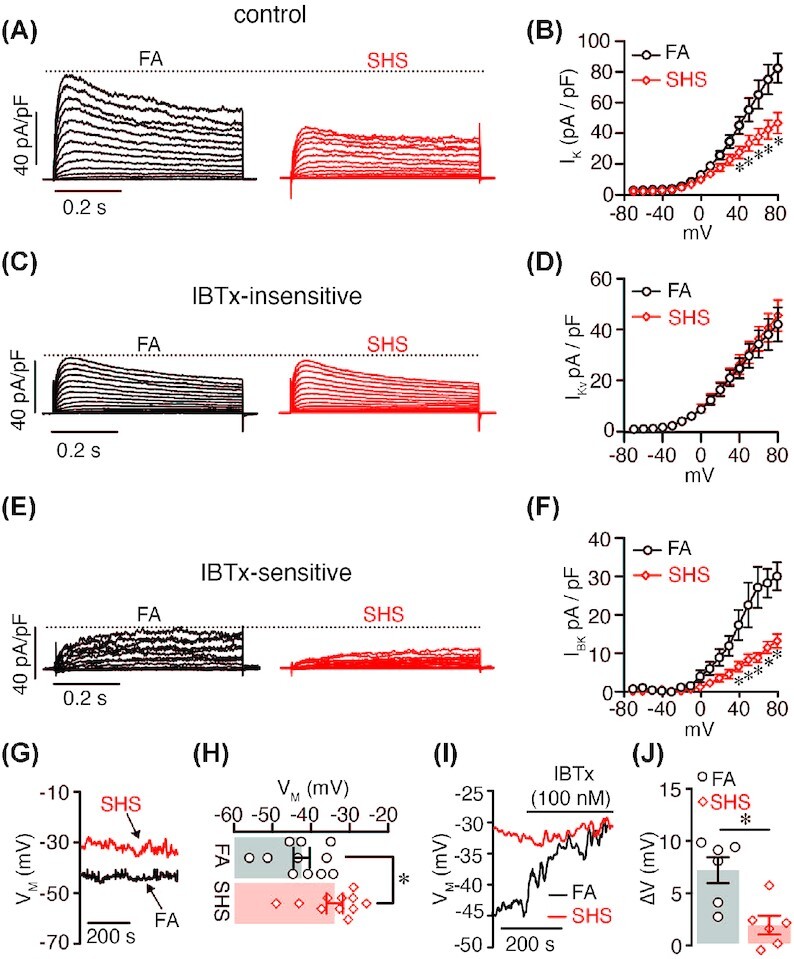
K^+^ channels in FA and chronic SHS-exposed arterial myocytes. (A–F) Representative whole-cell I_K_ (A), I_Kv_ (B), and I_BK_ (C) elicited by 10 mV depolarizing pulses from −70 to +80 mV and corresponding current–voltage relationship (B,D, and F) recorded from FA (*n* = 8 cells, 6 mice) and 12 wk SHS-exposed (*n* = 10 cells, 7 mice) mesenteric arterial myocytes. I_K_ was recorded before (A) and after (C) application of the BK_Ca_ channel inhibitor IBTx. The IBTx-sensitive component of the current (E) was obtained by digital subtractions of currents in the presence of IBTx from the currents before the application of the inhibitor. *P < .05; one-way ANOVA. (G) Representative *V_M_* traces recorded in current–clamp and (H) amalgamated data from FA (*n* = 11 cells, 6 mice) and 12 wk SHS-exposed (*n* = 11 cells, 5 mice) arterial myocytes (*P* < .05; *t*-test). (I) Representative *V_M_* traces recorded in current clamp and (J) scatter plot of IBTx-induced change in *V_M_* from FA (*n* = 6 cells, 4 mice) and 12 wk SHS-exposed (*n* = 6 cells, 4 mice) arterial myocytes before and after application of BK_Ca_ channel inhibitor IBTx (100 nM). *P* < .05; Mann–Whitney test.

K^+^ channels are key regulators of membrane potential and LTCC activity in arterial myocytes.^11^^,^^35^ Accordingly, activation of K^+^ channels lead to K^+^ efflux, membrane potential hyperpolarization and vasorelaxation. Thus, we examined K^+^ channel function in mesenteric arterial myocytes from FA and chronic SHS mice by measuring whole-cell K^+^ currents (I_K_) under voltage–clamp conditions. I_K_ were triggered by 200 ms depolarizing pulses from −70 mV to +80 mV in +10 mV step increments. Initial recordings revealed a significant reduction in I_K_ at several membrane potentials in arterial myocytes form SHS-exposed mice (Figure 4A and B). The peak I_K_ recorded at +80 mV was reduced by ∼56% in SHS-exposed arterial myocytes compared to FA cells (80.4 ± 10 in SHS vs 44.8 ± 6 pA in FA; * *P* < .05).

I_K_ in arterial myocytes predominantly result from activation of voltage-gated K^+^ (K_V_) and large-conductance Ca^2+^-activated K^+^ (BK_Ca_) channels.^11^^,^^35^ To determine if a decrease in K_V_, BK_Ca_ or both conductance contribute to reduced I_K_ in chronic SHS-exposed myocytes, we used a well-established pharmacological approach to separate the K_V_ (I_Kv_) and BK_Ca_ (I_BK_) currents.^36^^,^^37^ For this, we first recorded the whole-cell I_K_ as described above, followed by application of the highly selective BK_Ca_ channel inhibitor iberiotoxin (IBTx; 100 nM)^38^ to identify the IBTx-sensitive and IBTx-insensitive components reflecting I_BK_ and I_Kv_, respectively. Results showed no changes in the IBTx-insensitive component between FA and chronic SHS-exposed cells (Figure 4C and D), suggesting that I_Kv_ are not altered in mesenteric arterial myocytes exposed to chronic SHS. However, a significant decrease in the IBTx-sensitive component was observed in chronic SHS-exposed arterial myocytes compared to FA cells (Figure 4E and F). Peak I_BK_ at +80 mV was 14.0 ± 2.0 pA/pF in SHS cells and 30.8 ± 4.0 pA/pF in FA cells (*P* < .05). These results suggest that exposure to chronic SHS reduces I_BK_ in mesenteric arterial myocytes. Altogether, data suggest a distinctive reduction in I_BK_ but not I_Kv_ in mesenteric arterial myocytes exposed to chronic SHS.

To test the functional implications of reduced I_BK_ in chronic SHS-exposed arterial myocytes, membrane potential was measured using the perforated modality of the whole-cell patch-clamp technique in the current–clamp configuration using FA and chronic SHS cells. First, it was found that chronic SHS-exposed arterial myocytes had a more depolarized resting membrane potential (V_M_) compared to FA cells (−43.2 ± 2 mV in FA vs −35 ± 2 mV in SHS, *P* < .05: Figure 4G and H). Subsequently, IBTx elicited V_M_ depolarization of ∼7 ± 1 mV in FA cells, but only ∼2 ± 1 mV in chronic SHS cells (*P* < .05; Figure 4I and J). These results are consistent with reduced I_BK_ in chronic SHS arterial myocytes. Moreover, data indicate that compromised BK_Ca_ channel function may contribute to alter V_M_ and cell excitability in mesenteric arterial myocytes after chronic SHS exposure.

### BK_Ca_ Subunit Expression and NFAT Activation in Chronic SHS-Exposed Arterial Myocytes

**Figure 5.**
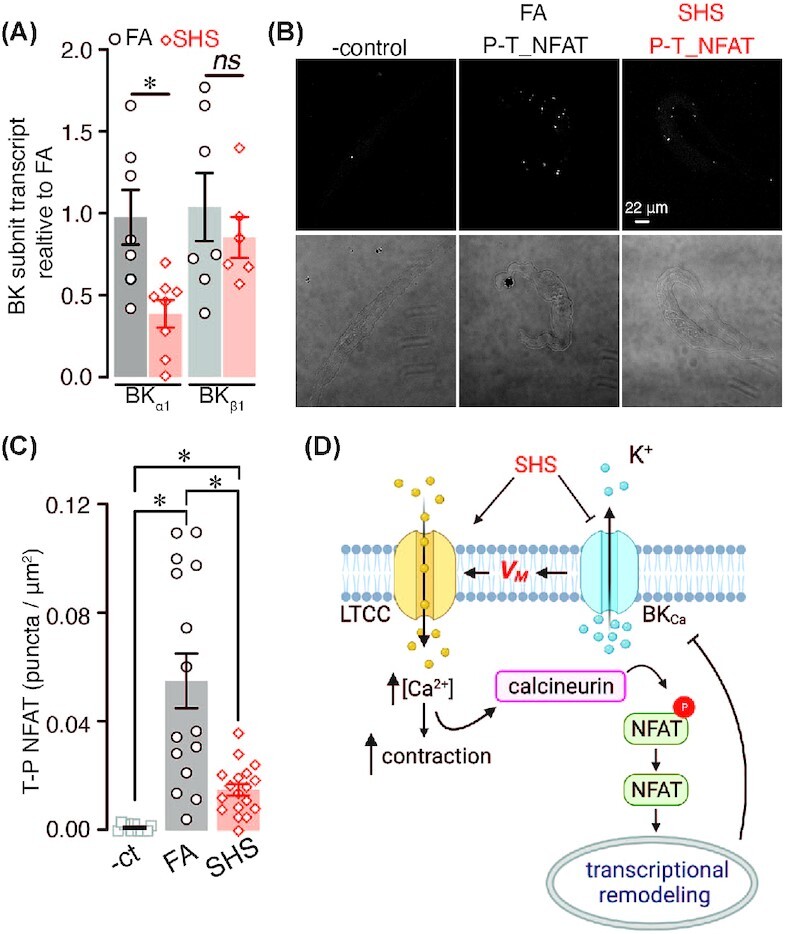
BK_Ca_ channel expression and NFAT activation state in FA and chronic SHS-exposed arterial myocytes. (A) Scatter plot of BK_Ca_ α and β1 subunits transcript expression in arteries from FA (*n* = 7 lysates; 7 mice) and 12 wk SHS-exposed (*n* = 6 lysates; 6 mice) mice (*P* < .05; Kruskal–Wallis test). (B) Representative fluorescence PLA (top) and differential interference contrast (bottom) images in FA and 12 wk SHS-exposed arterial myocytes labeled with phospho-NFAT and total-NFAT antibodies. (C) Quantification of PLA puncta per cell area (puncta/µm^2^) in arterial myocytes from mice exposed to FA (*n* = 15 cells) and SHS (*n* = 18 cells) (negative control *n* = 11 cells) (*P* < .05; Kruskal–Wallis, followed by Dunn's post-test). (D) Model of alterations in LTCC and K^+^ channels in mesenteric arterial myocytes exposed to SHS.

To examine mechanisms underlying chronic SHS-induced I_BK_ reduction in arterial myocytes, we quantified the expression of the BK_Ca_ channel pore-forming α subunit and accessory β1 subunit in response to FA and chronic SHS treatment. Quantitative PCR analysis revealed a significant reduction in total expression of the BKα1 subunit with no change in total expression of the accessory BKβ1 subunit in chronic SHS compared to FA samples (Figure 5A). These results suggest that downregulation of the BKα1 subunit may contribute to the reduction in I_BK_ observed in SHS-exposed mesenteric arterial myocytes.

Expression of BK α and β1 subunits is regulated by the transcription factor of activated T cells (NFATc3).^39^^,^^40^ NFAT activation requires its dephosphorylation by the phosphatase calcineurin (CaN) to unmask nuclear localization signals.^41^ The NFAT phosphorylation state can then serve as an indication of the activity of the transcription factor. Thus, to examine whether NFAT is activated in arterial myocytes in response to chronic SHS exposure, we used PLA as a proxy with antibodies against phosphorylated NFAT and total NFAT in FA and chronic SHS cells. The number of PLA puncta reflects NFAT activation state, with lower number of puncta indicating NFAT activation. This analysis found that the number of PLA puncta generated between the phosphorylated and total NFAT antibodies was significantly smaller in chronic SHS-exposed arterial myocytes compared to FA cells (Figure 5B and C). These data suggest that NFAT activity is higher in mesenteric arterial myocytes exposed to chronic SHS. Accordingly, NFAT activation may underlie the downregulation of the BKα1 subunit leading to reduced I_BK_ and depolarized V_M_ of arterial myocytes challenged with chronic SHS.

## Discussion

In this study, we found that resistance mesenteric arteries from mice exposed to SHS for 12 wk have higher levels of myogenic tone than arteries from control FA exposed mice. We also found up-regulation of LTCC functional expression, which is consistent with increased contractility. Moreover, we found a significant reduction in BK_Ca_ channel function following 12 wk of exposure. The decreased BK_Ca_ channel function correlated with lower levels of expression of the BKα1 subunit. These results suggest that SHS impairs vascular tone in a time-dependent manner with more prolonged exposure, having a significant effect on the remodeling of ion channels involved in vascular smooth muscle contractility regulation.

LTCC activity is linked to fundamental processes of arterial myocyte function, such as excitation–contraction and excitation–transcription coupling. The activity of LTCC is necessary for VSM contraction, playing a crucial role in regulating of arterial diameter and blood pressure. Not surprisingly, stimuli that impact LTCC activity could have significant consequences on vascular function. In our study, LTCC activity was higher in VSM from mice exposed to SHS. Single-channel events in cells from SHS exposed mice showed a higher frequency of coupled events, which could amplify Ca^2+^ influx through LTCC, and activate pathological signaling in the cardiovascular system.^42^^,^^43^ Higher LTCC coupling is shown to contribute to vascular dysfunction in animal models of hypertension and diabetes^42^^,^^44^ by activating pathological calcineurin/NFAT signaling pathway, leading to changes in expression of K^+^ channels.^35^^,^^42^ Augmented LTCC activity in SHS exposed mice was directly correlated with an increase in the expression of the LTCC α subunit Ca_V_1.2 as revealed by WB and proximity ligation assay; the precise mechanism remains to be elucidated, however. Nevertheless, our results are consistent with research showing an effect of SHS components (eg nicotine, CO, and particulate matter) on LTCC.^45–48^ The data in this manuscript suggest that SHS alters myogenic tone, at least in part by impacting LTCC functional expression.

The activity of several potassium (K^+^) channels regulates VSM membrane potential, which significantly influences intracellular Ca^2+^ through the regulation of voltage-gated L-type Ca^2+^ channels.^11^ Our study shows a significant decrease in I_K_ in cells isolated from arteries of mice exposed to SHS. Exposure to SHS did not significantly change the IBTx-insensitive (K_V_) component of the potassium current. We observed a significant reduction in BK_Ca_ channel current amplitude (ie the IBTx-sensitive component of the current). Moreover, consistent with reduced BK_Ca_ channel function, we found that VSM from SHS exposed mice had depolarized membrane potential compared to control. When challenged with IBTx control, FA cells responded with the expected *V_M_* depolarization, whereas cells from the SHS group failed to respond, indicating a compromised BK_Ca_ function. Reduced BK_Ca_ current was accompanied by lower expression of the BK channel alpha subunit. Our results are consistent with an effect of SHS on BK_Ca_ channels; combined with increased LTCC activity; this could lead to higher Ca^2+^ influx and impact contractility and transcriptional regulation. Indeed, VSM from SHS exposed mice co-labeled for total-NFAT and p-NFAT showed decreased PLA puncta indicating higher NFAT activity. These findings suggest a potential activation of the NFAT signaling pathway, which is consistent with previous studies showing increase LTCC activity leads to NFAT activation and transcriptional remodeling in smooth muscle cells, including BKα1 subunit expression.^39^^,^^40^

This study shows that exposure to an environmentally relevant SHS concentration impacts ion-channel function and arterial contractility in a time-dependent manner. We study only one SHS concentration. Future studies should assess longer timepoints and other SHS concentrations to better characterize the time- and concentration-dependent effects of SHS on smooth muscle remodeling underlying changes in mesenteric artery reactivity. Multiple components of tobacco smoke can contribute to its toxicity, including acute and chronic effects of nicotine, particulate matter load, and toxicant exposure. Further studies are necessary to elucidate whether nicotine, particle load, or both contribute to the observed remodeling of ion channels.

Recent population studies suggest a positive association between SHS exposure and hypertension.^49^ However, the mechanisms linking SHS to chronic hypertension are poorly understood. Our study may contribute to clarify this knowledge gap. Accordingly, we found increased myogenic tone in resistance size mesenteric arteries from 12 wk SHS-exposed mice (Figure 1). At the cellular level, increased myogenic tone was associated with high LTCC activity and decreased BK_Ca_ channel function in SHS-exposed arterial myocytes. Moreover, SHS-exposed mesenteric arteries showed increased activity of the transcription factor NFAT (Figure 5), which may contribute to transcriptional remodeling in these blood vessels. These results are the first to correlate changes in the activity of LTCC and BK_Ca_ channels and NFAT signaling to altered myogenic tone in response to SHS exposure. Because mesenteric arteries contribute to total peripheral resistance, changes in arterial myocyte electrophysiology and myogenic tone could represent a mechanism for increased blood pressure as well as a contributing factor for other vascular complications in people exposed to SHS.
